# Practical Medicine and Therapeutics

**Published:** 1837-01

**Authors:** 


### PRACTICAL MEDICINE AND THERAPEUTICS.

*On Typhus Fever in Britain compared with that in Paris and Geneva.*
By Dr. H. C. Lombard, of Geneva.

Dr. Lombard states that he came to this country after having studied fever both in Paris and Geneva for seven years, fully convinced that, in all cases of continued fever, there was enlargement or ulceration of the mucous glands of the small intestines; but, to his surprise, cases fell under his own observation, both in Glasgow and Dublin, in which there was no lesion whatever of the intestinal tube. The general symptoms which he had almost always seen in fever in Paris and Geneva were exactly those which he observed in Dublin and Glasgow, so that he was firmly convinced it was the same disease. The most obvious differences were, in the fevers of this country, greater quantity of the eruption of rosy spots which is always seen, but not in any extent, in continental typhus; and the occurrence of the disease in infants and very old people, which is not witnessed in France and Switzerland. Diarrhœa also, which is almost a constant symptom abroad, is less frequent in the fever of Dublin and Glasgow, which is also more highly contagious. These facts, which were very startling to Dr. Lombard, led him to modify his previous opinion that fever was connected with a diseased state of the intestinal canal, and to conclude that "typhus fever is more a general disease affecting the whole constitution, than a malady depending on any local inflammation, or any local change of structure." The practical results from this view of the subject are, that one mode of treatment is not universally applicable, and that charity should be exercised when one set of pathologists criticise another in a different country.

In a subsequent communication written at Geneva, after reflecting on these facts and seeing cases of fever in England, Dr. Lombard endeavours to reconcile some of these differences. In the Fever Hospital of Liverpool he saw a hundred cases resembling those in Dublin very closely. In Manchester Fever Hospital, the proportionate number of cases was much less, but the symptoms very similar. In Birmingham there was no fever hospital, and but one fever ward in the Infirmary: he was told that fever cases were by no means frequent, but that, where they proved fatal, ulcerations in the ileum were always found. In the London Fever Hospital there were only twelve cases; the symptoms were similar to the Dublin typhus, and, according to Dr. Tweedie's researches, ulceration of the intestines were not found in more than one-fourth of the cases. From these facts Dr. Lombard adopts the opinion that Ireland is the source of the contagious fever which prevails in Great Britain, and that it is the same disease which the French have called "*typhus contagieux, fièvre des armées, fièvre des prisons*," as described in 1813, 14, 15, when it prevailed wherever the armies met and sojourned. In twenty-five years there have been 77,866 cases in the Cork-street Fever Hospital in Dublin. Wherever the Irish poor go they carry this fever: hence it is prevalent in Glasgow and Liverpool, where the Irish pass in great numbers; and less so in Manchester, Birmingham, and London. But Dr. Lombard does not regard this as the only source of fever: fever is also sporadic, and he believes that this sporadic form is the common continued fever of the country, always attended with disease of the mucous glands of the small intestines, and similar to the typhoid or continued fever of Paris and Geneva.

[This paper of Dr. Lombard's is of much importance, although the first part of it contains nothing new, the opinions being exactly similar to those commonly embraced in this country. For, notwithstanding Dr. Alison and other pathologists have stated most emphatically that follicular disease of the intestines was not universal in the typhoid fever of this country, the fact has not met with the attention which so high an authority should command, from its being in direct contradiction to the constant experience of the Paris pathologists. The testimony of one who confesses his previous scepticism may be of greater service in producing conviction. The theory with which Dr. Lombard concludes is very ingenious: it may be that the highly contagious typhus of Dublin and Glasgow is identical with the camp typhus of 1814, described by Chomel and others; but we do not think that Dr. Lombard has sufficient facts to prove that the common continued fever of this country is always attended with follicular disease, as in Paris. We are very deficient in exact and extensive information on this point.] *Dublin Journal, September,* 1836.

---

*Case of Spectral Illusions, with Loss of the Memory of Words.*
By JAMES CRAIG, Esq., Surgeon, Ratho.

A GENTLEMAN, (designated Mr. N.,) of very superior mental endowments, retired from public life in the seventy-seventh year of his age, when still in good health. He could speak ten languages, and read fourteen. His habits were temperate. In 1819, when seventy-six years old, he began to have almost daily visitations of spectral images, generally of human faces and upper parts of the body, as large as life, and defined with the clearness and minute finishing of the finest painting; the lower parts were lost in a shadowy mist. The costumes were of various nations. He could never trace any resemblance of the faces to persons he had known: his own countenance, however, was occasionally presented to him, undergoing the change from youth to manhood, and from manhood to age. The figures were seen equally well with the eyes shut or open, in daylight or in darkness: they were always agreeable; he could call them up at will, and banish them by drawing the hands over the eyes, but they often speedily reappeared. In August 21st, 1832, he was suddenly deprived of the power to pronounce articulate language, whilst sitting at dinner; but in an hour afterwards he talked quite well, but in a slower tone. Previously to this he had suffered anxiety and annoyance, and his memory had become impaired, so that he could not recollect the names of his friends or of the plants in his conservatory. The next day he had a similar attack, and his conversation afterwards was generally unintelligible; in one sentence you might recognize French, Italian, Spanish, and but very rarely any English words; sometimes mere gibberish. On certain occasions, as when his feelings were strongly excited, or when he was suddenly surprised, or when he was roused to make an effort, he spoke a sentence in English distinctly and accurately. He was evidently quite conscious of what was going on, and understood perfectly what was said to him; he also recollected places, persons, and objects. On one occasion he imagined he saw his wife, (who had been dead several years,) beckoning him to follow her out of the window. The hallucination was so vivid, that he jumped out at the window, (a height of seven and a half feet,) and fell on the grass; but the fall did not dissipate the delusion. He related the particulars, chiefly in English, to his overseer, of whom he enquired if he had seen his wife. In this condition he continued four years. In July, 1836, he caught cold, which was followed by fever, under which he laboured nine days, and gradually sank, evincing to the last the same degree of sensibility he had done for years. Death took place in the ninety-third year of his age.

On examination, the dura mater adhered strongly to the skull; considerable effusion and vascularity over the surface of the brain, indicating chronic inflammation of the membranes, especially in the course of the superior longitudinal sinus, where the dura mater was very thick. Right hemisphere healthy, with the exception of a small tubercle near the surface. In the posterior lobe of the left hemisphere was a cavity, exceeding two inches in length, running outside of the left lateral ventricle, lined with a yellowish membrane, and the brain around it was much softened. In the middle lobe, a little behind the pituitary gland, was a similar diseased appearance, not exceeding a quarter of an inch in extent. The membranes of the base of the brain bore strong marks of chronic inflammation. Cerebellum sound. A small tubercle on the posterior part of the medulla oblongata. Semilunar valves of the aorta ossified.

[The paper from which we have taken the above particulars is very interestingly drawn up, and is followed by important pathological remarks by Dr. Craigie. We subjoin brief notices of cases somewhat analogous: the first (*a*,) recorded in Mr. Craig's paper, by Dr. Davidson, one of Mr. N.'s medical attendants; the other (*b*,) extracted from a communication of Dr. Lendrick's, in the Dublin Journal for September, 1836.]

(*a*.) In the Eloges of Cuvier it is mentioned that Broussonet's memory of language was affected in a singular manner. He had a slight attack of apoplexy, but of one faculty no restoration took place. He was never able to pronounce or write accurately substantive nouns and proper names, either in French or Latin, though he had complete power over the other parts of these languages. Epithets and adjectives he could accumulate in a manner sufficiently striking to make himself understood. When he wished to designate any individual, he recalled his figure, his qualities, and his occupations. Upon inspecting the head, there was found on the surface of the brain, on the left side, a large ulcer partly cicatrized.

(*b.*) A gentleman, distinguished in Trinity College, Dublin, for his scientific attainments, became paralytic, and afterwards his memory was so impaired that he recollected imperfectly only two or three friends. He had completely forgotten the use and nature of letters, and could neither write nor read a syllable, nor even comprehend the alphabet. He, however, perfectly recollected the use of figures, and could solve the most intricate arithmetical problems; but, if algebraical symbols were introduced, (and he had been a distinguished algebraist,) the connexion with letters confused him, and he was completely at fault.

*Edinburgh Journal, October*, 1836.

---

*On Dropsy following Scarlet Fever.* By J. Stark, m.d., Edinburgh.

Dr. Stark has given an excellent account of the scarlet fever as it prevailed epidemically in Edinburgh, in the autumn and winter of 1835-36. In fifteen cases dropsical sequelæ followed. In the most severe cases, the swelling came on suddenly without any previous complaint, and from a fortnight to three weeks after the disappearance of the eruption. The patient went to bed well, and in the night or the next morning the friends were alarmed by the sudden swelling of the body, attended with dyspnœa, moaning, restlessness, and sometimes stupor. In all the cases in which the urine was examined, it was found coagulable by heat. In many of the severer cases, the urine passed before the dropsy came on was very turbid, dark coloured, in a few cases bloody. In two cases it was suppressed. Exposure to cold seemed the immediate cause of the dropsy: the function of the skin being checked, the blood is thrown upon the internal organs, and the kidney, whose action is vicarious with the skin, particularly suffers.

In all cases, except the very mildest, he bled in proportion to the severity of the symptoms, and the relief experienced from it. In severe cases he bled from the arm until the pulse was affected: under two years of age, leeches were used. The bleeding was followed by small doses of the antimonial wine with the liquor ammon. acet., and, where the urine was suppressed, a large hot bran poultice was applied to the lumbar region. In milder cases, the same medicines with brisk purging. The warm bath was of the utmost advantage. There was but one fatal case, of a boy five and a half years old, whose parents would not permit bleeding or leeches. Dissection was not allowed.

[The explanation of the action of cold in producing dropsy is given by Dr. Stark as his own, on which he founded his practice, which was very successful. It is but justice, however, to Dr. Osborne to state that he had previously advanced an identical explanation. (See British and Foreign Medical Review, vol. ii. p. 222.)]

*Edinburgh Medical and Surgical Journal, October*, 1836.

---

*On distinguishing Neuralgic from Inflammatory Affections.*
By W. Griffin, m.d., Limerick.

Dr. Abercrombie has laid it down that, in peritoneal and enteric inflammations, the most important symptom is tenderness of the abdomen; and that, whenever this occurs, whatever may be the state of the bowels or pulse, or complaint of pain, the case should be most anxiously watched. There can be no doubt, however, that, although tenderness on pressure always accompanies inflammation, yet it may be present without inflammation, and such cases are very puzzling to the young practitioner. Dr. Griffin offered a suggestion several years since as a result of his experience, which has not been sufficiently attended to, and he now illustrates it by several cases. He then stated that, in any doubtful cases, if there was tenderness at a portion of the spinal cord corresponding with the disturbed organ, that it might be considered decidedly neuralgic; but, if no such symptom was found, it was probably inflammatory. This is applicable to the neuralgic pains of the chest simulating pleurisy, as well as to abdominal affections. Three cases are given. The treatment does not seem to have been directed to the spine itself, but to the bowels—opium, fomentations, and purgatives. The patients were women.

*Dublin Journal, September*, 1836.

---

*Cases illustrative of the Effects of the Saline Treatment in Morbid Conditions of the Blood.* By C. R. BREE, Esq., Stowmarket.

THE mode of treatment introduced by Dr. Stevens has been cried up and cried down too much, and we still want sufficient evidence to enable us to decide on its merits. Mr. Bree's communication contains three cases, two of typhus fever and one of purpura. In all the cases amendment took place shortly after the administration of the remedy, but it is not so clear that these two events stood in the relation of cause and effect. In the first case, an ounce of wine was given every two hours, contemporaneously with the saline powder; and the benefit might certainly be owing to the former. In the second case, there is no proof of diseased blood, nor indeed of great severity of symptoms. The case of purpura occurred in an infant in an aggravated form, and there seems every reason to conclude that the saline treatment was most effective in removing the disease. The following is the formula of the remedy given to the child, (æt. fifteen months:)

℞. Sod. Carb. ℈ij.; Sod. Mur. ʒss.; Pot. Chlor. gr. x.; Syr. Ros. ℥ss.; Aquæ, ℥i. M. coch. parv. ter die.

We hope Mr. Bree will proceed with his experiments.

*Lancet, October* 1, 1836.

---

*Cases of Spasm of the Muscles of the Neck, sympathetic with Disorder in other Parts.* By R. HUTCHINSON, M.D., Nottingham.

UNDER this title Dr. H. details three cases, which appear to him "to proceed from a similar cause, viz. morbid irritation affecting the *nervus accessorius ad p. octavum*, either arising immediately from the base of the brain itself, or from the upper portion of the medulla spinalis, or indirectly reflected from disordered function in distant organs, and, above all, from a deranged condition of the stomach."—"The most prominent condition is a spasm, more or less constant, of either one or both trapezius muscles, or of one sterno-cleido mastoideus."—"The spasm is attended with considerable pain and apprehension, so much so, that the patient dreads walking, which excites it considerably." The spasms abate at night, and the sleep is undisturbed, but during the day they continue with but little intermission. The cases were treated variously, according to the supposed source of the irritation producing the spasm. They proved all very obstinate. *Lancet, September* 24, 1836.

---

*On the Nature and Treatment of Acute Neuralgia.* By F. C. SKEY, Esq.

MR. SKEY argues against the opinion, which it appears is held by some persons, that neuralgia is an inflammatory disease. He adduces several cases to show that it is not relieved by local depletory means and counter-irritation, and others to show the beneficial effects of muriate of ammonia (in doses of a scruple or half a drachm), colchicum, and aconitine. Two cases are related in which this last medicine, locally applied, in the proportion of one grain to the drachm of cerate, was productive of striking and permanent relief in tic douleureux.

*Medical Gazette, November* 5, 1836.

---

BARON SLOET'S *Method of treating Epilepsy.*

THIS is communicated by Dr. Aldis, to whom the Baron made known the secret, which had been preserved in his family for more than two centuries. The remedy is said to have been singularly successful, though we fear its virtues will vanish with its publicity; it is as follows:—℞. Cort. Rad. Dictammi albi, (Fraxinella,) lb j.; Pulv. Zedoariæ, ℥iss. The dose is about two scruples from once to four times daily.

*Medical Gazette, October* 29, 1836.

---

*Influence of Mental on Bodily Functions.* By C. O'Reilly, m.d. Dublin.

A healthy young woman, of a nervous temperament, was extremely affected on hearing of the death of her brother, and declared she could not survive him one week. She retired to bed, refused all sustenance for three days: on the evening of the fourth day she took a little food, which she vomited. She died during the night. The body was examined, but no morbid condition was detected. Several similar cases are referred to: hundreds might be added. *Lancet, Nov.* 5, 1836.

---

*On the Use and Abuse of Aloes.* By E. Greenhow, m.d., North Shields.

The object of the author of this paper seems chiefly to show, what all experienced practitioners know, that aloes, if long continued, produces irritation of the bowels, and particularly of the rectum; and that small doses are better than large. He says that from two to five grains will be sufficient: he might have said that half a grain, or even a quarter of a grain, will often be so, if minutely comminuted with mastich and rose-leaves, or many other inert substances. Dr. G. says that the addition of ipecacuan has the effect of diminishing or removing its irritating effect on the anus; and that its addition to squill and other diuretics often greatly promotes their action.

*Medical Gazette, November* 19, 1836.

---

St. John Long's *Liniment.*

Mr. Guthrie, having had presented to him, for the purpose of trying its effects, some of the once famous liniment of Mr. Long, selected some cases for its application, and also had it applied to his own person, he being affected at the same time with a pain in the knee, attended with slight lameness. The experiment was conducted openly at the Ophthalmic Hospital, the liniment being applied by Mr. Wood, the person who rubbed under Long. It was used in five cases, besides Mr. Guthrie's own, but the disease of one only is stated, viz. that of a boy who is said "to have come up amaurotic from the country." The result of the treatment is thus given by Mr. Guthrie: "It cured my knee and the boy's eye, and did good to all the remaining four." The liniment appears to be perfectly mild and harmless, looking like thick yellow cream, and having a faint turpentine smell. Applied to the skin, it felt cool and agreeable, and not in the slightest degree stimulating. It was assiduously rubbed on the part by means of a small, soft, round sponge; and, after a sufficient application, the part became red, and finally excoriated and inflamed. Mr. Guthrie attributes the whole effect of the liniment to the mode of its application, and nothing to its own virtues. In proof of this, he had himself rubbed with soap-suds in the same manner as was done with the liniment, and exactly the same result followed. "I should have said, if I had been asked," says Mr. Guthrie, "that the soap-lather was the most severe liniment of the two."

[The mystery of St. John Long's operations, and of his (doubtless) occasional success, seems thus cleared up; and we consider the profession much indebted to Mr. Guthrie for its solution. We do not doubt that this particular mode of counter-irritation may be very advantageously applied in many cases both of acute and chronic diseases.] *Lancet, November* 26, 1836.

